# A list of bees from three locations in the Northern Rockies Ecoregion (NRE) of western Montana

**DOI:** 10.3897/BDJ.6.e27161

**Published:** 2018-10-30

**Authors:** Elizabeth G. Reese, Laura A. Burkle, Casey M. Delphia, Terry Griswold

**Affiliations:** 1 Department of Ecology, Montana State University, Bozeman, United States of America Department of Ecology, Montana State University Bozeman United States of America; 2 USDA-ARS Pollinating Insects Research Unit, Logan, United States of America USDA-ARS Pollinating Insects Research Unit Logan United States of America

**Keywords:** Apoidea, wild bees, biodiversity, pollinator, Montana, Rocky Mountains, Northern Rockies Ecoregion

## Abstract

**Background:**

Wild bees that were collected in conjunction with a larger study are presented as a checklist of species for the Northern Rockies Ecoregion of Montana, USA. Over the course of four field seasons (2013-2016), 281 species and morphospecies in 32 genera and five families were collected using insect nets, and identified. This paper addresses the distinct lack of studies monitoring bee species in Montana and contributes to a basic understanding of fauna in the northern Rocky Mountains.

**New information:**

With this study, the number of known bee species in Montana increases by at least six species, from 366 (Kuhlman and Burrows 2017) to 372. Though literature was not reviewed for all the species on this checklist, published records in Montana revealed no listings for *Andrena saccata* Viereck; *Anthidiellum notatum robertsoni* (Cockerell); *Ashmeadiella meliloti* (Cockerell); *Ashmeadiella pronitens* (Cockerell); *Colletes lutzi lutzi* Timberlake; and *Dioxys productus* (Cresson).

## Introduction

Faunistic studies characterizing native pollinators are becoming more important for understanding patterns in local and regional biodiversity as species are threatened by habitat loss, climate change and other factors (Green et al. 2005, Lengyel et al. 2008, Losos et al. 2013). Studies documenting wild bee species are particularly critical because bees are the world's primary animal pollinators in most ecosystems (Neff and Simpson 1993, Winfree 2010) and are currently undergoing declines across different spatial scales (Koh et al. 2015), including globally (Council, Natural Resource 2007). Much taxonomic work has focused on regional bee fauna in the eastern United States and several states in the western U.S., however there remains a dearth of studies documenting wild bees in Montana (but see Dolan et al. 2017 and Kuhlman and Burrows 2017).

Here we present a list of bee species from three localities in the northern Rocky Mountains collected over the course of four years as part of a larger project investigating the effects of wildfire on plant-pollinator diversity (Burkle et al. 2015). This paper contributes to the regional list of wild bees in Montana and addresses the need for pollinator monitoring in the western U.S.

## Materials and methods

### Study Region

**Figure 1.**
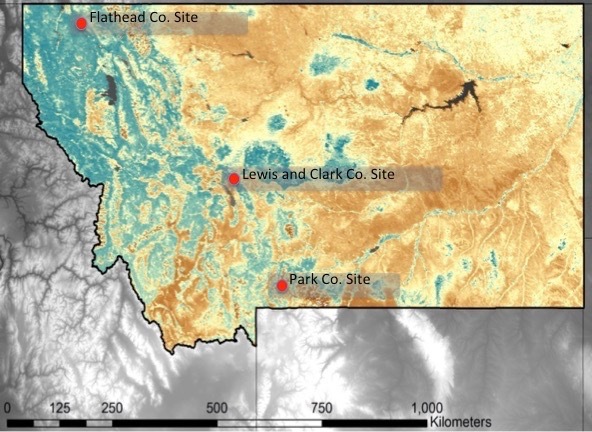
Map of study localities in the Northern Rockies, Montana, USA.

The Northern Rockies Ecoregion (NRE) encompasses 162,746 km^2^ of Idaho, western Montana and northeastern Washington, and is characterized by high, rugged mountain ranges consisting of montane, alpine and subalpine ecosystems (Taylor 2012). Dominant vegetation includes lodgepole pine (*Pinus contorta*), ponderosa pine (*Pinus ponderosa*), western larch (*Larix occidentalis*), and mixed conifer forests. Climate in the NRE is dry, with most precipitation occurring as snow, and with very cold winters and short summers (Table 1). We collected bee specimens from the following three localities within the NRE: near Glacier National Park in Flathead County, Absaroka-Beartooth Wilderness in Park County, and Helena National Forest in Lewis and Clark County (see Fig. 1).

### Collection Methods

At each locality, we established nine 25m diameter circular plots within each of four 15 hectare study blocks. Study blocks were located within previously determined wildfire perimeters. Within a block, plots were randomly stratified to meet research goals related to wildfire severity and to avoid spatial clumping of plots using a generalized random tessellation stratified (GRTS) survey design function in the R spsurvey package (Kincaid and Olsen 2011). Details of site selection and characteristics can be found in Burkle et al. 2015.

Bees were collected over four field seasons (2013-2016) beginning when plants started to bloom after snowmelt (late-May or June) and ending once most flower species had gone to seed (late-August). Collection dates for each species can be found in Supplementary Data Table 1 (Suppl. material 1). Each locality was sampled as often as possible per season, with sampling bouts being chiefly determined by weather. On every visit to a locality, we sampled each plot for 20 minutes. All insects that contacted the reproductive parts of flowers were collected with insect nets, pinned, and labeled with pertinent data, including plant species association. We visited the Lewis and Clark County locality five times in 2013, thirteen times in 2014, nine times in 2015 and seven times in 2016. At the Park County location, we visited two, eleven, nine, and six times in 2013, 2014, 2015 and 2016, respectively; and we visited the Flathead County location three times in 2013, and seven times in both 2014 and 2015.

### Species Identification

Bee species were identified by Elizabeth Reese, Terry Griswold, Casey Delphia, Skyler Burrows, Harold Ikerd, Michael Orr, Jason Gibbs and Karen Wright. To make determinations to the lowest possible taxonomic level we used published keys or unpublished works where these were available (Table 2). When keys were not sufficient, or did not exist for this area, we designated morphospecies based on morphological characteristics. Morphospecies were labeled "F" for female or "M" for male and given unique numbers. In cases where two particular species were impossible to differentiate based on morphology, both species names were notated (i.e., females of *Agapostemontexanus/angelicus*). Specimens are deposited in the Montana State University Pollinator Health Center Collection located in the Burkle Community Ecology Lab at Montana State University in Bozeman, MT and the U.S. National Pollinating Insect Collection in Logan, UT.

## Checklists

### List of Bees

#### Colletes consors consors

Cresson, 1868

##### Notes

Collected from the Lewis and Clark County site (Table 1, Suppl. material 1)

#### Colletes fulgidus

Swenk, 1904

##### Notes

Collected from the Lewis and Clark County and Park County sites(Table 1, Suppl. material 1)

#### Colletes hyalinus hyalinus

Provancher, 1888

##### Notes

Collected from the Park County site (Table 1, Suppl. material 1)

#### Colletes kincaidii

Cockerell, 1898

##### Notes

Collected from the Lewis and Clark County site (Table 1, Suppl. material 1)

#### Colletes lutzi lutzi

Timberlake, 1943

##### Notes

New species for Montana. Collected from the Lewis and Clark County and Park County sites (Table 1, Suppl. material 1)

#### Colletes phaceliae

Cockerell, 1906

##### Notes

Collected from the Lewis and Clark County site (Table 1, Suppl. material 1)

#### Hylaeus (Cephalylaeus) basalis

(Smith, 1853)

##### Notes

Collected from the Park County and Flathead County sites (Table 1, Suppl. material 1)

#### Hylaeus (Hylaeus) annulatus

(Linnaeus, 1758)

##### Notes

Collected from the Lewis and Clark County, Park County and Flathead County sites (Table 1, Suppl. material 1)

#### Hylaeus (Hylaeus) leptocephalus

(Morawitz, 1871)

##### Notes

Collected from the Lewis and Clark County site (Table 1, Suppl. material 1)

#### Hylaeus (Hylaeus) mesillae

(Cockerell, 1896)

##### Notes

Collected from the Lewis and Clark County site (Table 1, Suppl. material 1)

#### Hylaeus (Hylaeus) rudbeckiae

(Cockerell and Casad, 1895)

##### Notes

Collected from the Lewis and Clark County site (Table 1, Suppl. material 1)

#### Hylaeus (Hylaeus) verticalis

(Cresson, 1869)

##### Notes

Collected from the Lewis and Clark County, Park County and Flathead County sites (Table 1, Suppl. material 1)

#### Hylaeus (Paraprosopis) coloradensis

(Cockerell, 1896)

##### Notes

Collected from the Lewis and Clark County and Park County sites (Table 1, Suppl. material 1)

#### Hylaeus (Paraprosopis) nevadensis

(Cockerell, 1896)

##### Notes

Collected from the Lewis and Clark County site (Table 1, Suppl. material 1)

#### Hylaeus (Paraprosopis) wootoni

(Cockerell, 1896)

##### Notes

Collected from the Lewis and Clark County and Park County sites (Table 1, Suppl. material 1)

#### Hylaeus (Prosopis) episcopalis

(Cockerell, 1896)

##### Notes

Collected from the Lewis and Clark County and Park County sites (Table 1, Suppl. material 1)

#### Hylaeus (Prosopis) modestus

Say, 1837

##### Notes

Collected from the Lewis and Clark County, Park County and Flathead County sites (Table 1, Suppl. material 1)

#### Andrena (Andrena) milwaukeensis

Graenicher, 1903

##### Notes

Collected from the Lewis and Clark County and Park County sites (Table 1, Suppl. material 1)

#### Andrena (Andrena) saccata

Viereck, 1904

##### Notes

New species for Montana. Collected from the Park County site (Table 1, Suppl. material 1)

#### Andrena (Andrena) thaspii

Graenicher, 1903

##### Notes

Collected from the Lewis and Clark County, Park County and Flathead County sites (Table 1, Suppl. material 1)

#### Andrena (Andrena) topazana

Cockerell, 1906

##### Notes

Collected from the Park County and Flathead County sites (Table 1, Suppl. material 1)

#### Andrena (Andrena) sp. F1

##### Notes

Collected from the Lewis and Clark County site (Table 1, Suppl. material 1)

#### Andrena (Cnemidandrena) surda

Cockerell, 1910

##### Notes

Collected from the Lewis and Clark County site (Table 1, Suppl. material 1)

#### Andrena (Diandrena) evoluta

Linsley & MacSwain, 1961

##### Notes

Collected from the Lewis and Clark County site (Table 1, Suppl. material 1)

#### Andrena (Euandrena) lawrencei

Viereck, & Cockerell, 1914

##### Notes

Collected from the Lewis and Clark County site (Table 1, Suppl. material 1)

#### Andrena (Euandrena) nigrocaerulea

Cockerell, 1897

##### Notes

Collected from the Lewis and Clark County site (Table 1, Suppl. material 1)

#### Andrena (Geissandrena) trevoris

Cockerell, 1897

##### Notes

Collected from the Lewis and Clark County site (Table 1, Suppl. material 1)

#### Andrena (Melandrena) nivalis

Smith, 1853

##### Notes

Collected from the Lewis and Clark County and Flathead County sites (Table 1, Suppl. material 1)

#### Andrena (Melandrena) pertristis

Cockerell, 1905

##### Notes

Collected from the Lewis and Clark County site (Table 1, Suppl. material 1)

#### Andrena (Melandrena) transnigra

Viereck, 1904

##### Notes

Collected from the Lewis and Clark County site (Table 1, Suppl. material 1)

#### Andrena (Melandrena) vicina

Smith, 1853

##### Notes

Collected from the Lewis and Clark County site (Table 1, Suppl. material 1)

#### Andrena (Micrandrena) melanochroa

Cockerell, 1898

##### Notes

Collected from the Lewis and Clark County and Park County sites (Table 1, Suppl. material 1)

#### Andrena (Micrandrena) microchlora

Cockerell, 1922

##### Notes

Collected from the Lewis and Clark County site (Table 1, Suppl. material 1)

#### Andrena (Plastandrena) crataegi

Robertson, 1893

##### Notes

Collected from the Lewis and Clark County and Park County sites (Table 1, Suppl. material 1)

#### Andrena (Plastandrena) prunorum

Cockerell, 1896

##### Notes

Collected from the Lewis and Clark County and Park County sites (Table 1, Suppl. material 1)

#### Andrena (Scaphandrena) aff. shoshoni

Ribble, 1974

##### Notes

Collected from the Lewis and Clark County site (Table 1, Suppl. material 1)

#### Andrena (Scaphandrena) scurra

Viereck, 1904

##### Notes

Collected from the Lewis and Clark County site (Table 1, Suppl. material 1)

#### Andrena (Scaphandrena) walleyi

Cockerell, 1932

##### Notes

Collected from the Lewis and Clark County site (Table 1, Suppl. material 1)

#### Andrena (Thysandrena) candida

Smith, 1879

##### Notes

Collected from the Park County and Flathead County sites (Table 1, Suppl. material 1)

#### Andrena (Thysandrena) knuthiana

Cockerell, 1901

##### Notes

Collected from the Park County and Flathead County sites (Table 1, Suppl. material 1)

#### Andrena (Thysandrena) medionitens

Cockerell, 1902

##### Notes

Collected from the Lewis and Clark County site (Table 1, Suppl. material 1)

#### Andrena (Thysandrena) vierecki

Cockerell, 1904

##### Notes

Collected from the Park County site (Table 1, Suppl. material 1)

#### Andrena (Trachandrena) amphibola

(Viereck, 1904)

##### Notes

Collected from the Lewis and Clark County, Park County and Flathead County sites (Table 1, Suppl. material 1)

#### Andrena (Trachandrena) cleodora

(Viereck, 1904)

##### Notes

Collected from the Lewis and Clark County and Flathead County sites (Table 1, Suppl. material 1)

#### Andrena (Trachandrena) cupreotincta

Cockerell, 1901

##### Notes

Collected from the Lewis and Clark County site (Table 1, Suppl. material 1)

#### Andrena (Trachandrena) miranda

Smith, 1879

##### Notes

Collected from the Lewis and Clark County and Park County sites (Table 1, Suppl. material 1)

#### Andrena (Trachandrena) salicifloris

Cockerell, 1897

##### Notes

Collected from the Lewis and Clark County and Park County sites (Table 1, Suppl. material 1)

#### Andrena (Trachandrena) sigmundi

Cockerell, 1902

##### Notes

Collected from the Lewis and Clark County site (Table 1, Suppl. material 1)

#### Andrena sp. F7

##### Notes

Collected from the Lewis and Clark County and Park County sites (Table 1, Suppl. material 1)

#### Andrena sp. F8

##### Notes

Collected from the Lewis and Clark County and Flathead County sites (Table 1, Suppl. material 1)

#### Andrena sp. F11

##### Notes

Collected from the Lewis and Clark County site (Table 1, Suppl. material 1)

#### Andrena sp. F12

##### Notes

Collected from the Lewis and Clark County site (Table 1, Suppl. material 1)

#### Andrena sp. F13

##### Notes

Collected from the Lewis and Clark County site (Table 1, Suppl. material 1)

#### Andrena sp. F14

##### Notes

Collected from the Lewis and Clark County site (Table 1, Suppl. material 1)

#### Andrena sp. F15

##### Notes

Collected from the Park County site (Table 1, Suppl. material 1)

#### Andrena sp. F16

##### Notes

Collected from the Lewis and Clark County site (Table 1, Suppl. material 1)

#### Andrena sp. F17

##### Notes

Collected from the Lewis and Clark County and Park County sites (Table 1, Suppl. material 1)

#### Panurginus atriceps

(Cresson, 1878)

##### Notes

Collected from the Lewis and Clark County, Park County and Flathead County sites (Table 1, Suppl. material 1)

#### Panurginus sp. F1

##### Notes

Collected from the Park County site (Table 1, Suppl. material 1)

#### Panurginus sp.1

##### Notes

Collected from the Park County site (Table 1, Suppl. material 1)

#### Protandrena (Pterosarus) innuptus

(Cockerell, 1896)

##### Notes

Collected from the Lewis and Clark County site (Table 1, Suppl. material 1)

#### Agapostemon (Agapostemon) texanus

Cresson, 1872

##### Notes

Collected from the Lewis and Clark County site (Table 1, Suppl. material 1)

#### Agapostemon (Agapostemon) virescens

(Fabricius, 1775)

##### Notes

Collected from the Lewis and Clark County site (Table 1, Suppl. material 1)

#### Dufourea dilatipes

Bohart, 1948

##### Notes

Collected from the Park County site (Table 1, Suppl. material 1)

#### Dufourea maura

(Cresson, 1878)

##### Notes

Collected from the Lewis and Clark County, Park County and Flathead County sites (Table 1, Suppl. material 1)

#### Dufourea trochantera

Bohart, 1948

##### Notes

Collected from the Lewis and Clark County site (Table 1, Suppl. material 1)

#### Halictus (Nealictus) farinosus

Smith, 1853

##### Notes

Collected from the Lewis and Clark County site (Table 1, Suppl. material 1)

#### Halictus (Odontalictus) ligatus

Say, 1837

##### Notes

Collected from the Lewis and Clark County site (Table 1, Suppl. material 1)

#### Halictus (Protohalictus) rubicundus

(Christ, 1791)

##### Notes

Collected from the Lewis and Clark County, Park County and Flathead County sites (Table 1, Suppl. material 1)

#### Halictus (Seladonia) confusus

Smith, 1853

##### Notes

Collected from the Lewis and Clark County, Park County and Flathead County sites (Table 1, Suppl. material 1),)

#### Halictus (Seladonia) tripartitus

Cockerell, 1895

##### Notes

Collected from the Lewis and Clark County site (Table 1, Suppl. material 1)

#### Lasioglossum (Dialictus) abundipunctum

Gibbs, 2010

##### Notes

Collected from the Lewis and Clark County site (Table 1, Suppl. material 1)

#### Lasioglossum (Dialictus) aff.caducum

(Sandhouse, 1924)

##### Notes

Collected from the Lewis and Clark County site (Table 1, Suppl. material 1)

#### Lasioglossum (Dialictus) aff.nevadense

(Crawford, 1907)

##### Notes

Collected from the Lewis and Clark County site (Table 1, Suppl. material 1)

#### Lasioglossum (Dialictus) albipenne

(Robertson, 1890)

##### Notes

Collected from the Lewis and Clark County site (Table 1, Suppl. material 1)

#### Lasioglossum (Dialictus) brunneiventre

(Crawford, 1907)

##### Notes

Collected from the Lewis and Clark County site (Table 1, Suppl. material 1)

#### Lasioglossum (Dialictus) ebmerellum

Gibbs, 2010

##### Notes

Collected from the Lewis and Clark County site (Table 1, Suppl. material 1)

#### Lasioglossum (Dialictus) ephialtum

Gibbs, 2010

##### Notes

Collected from the Park County and Flathead County sites (Table 1, Suppl. material 1)

#### Lasioglossum (Dialictus) hudsoniellum

(Cockerell, 1919)

##### Notes

Collected from the Lewis and Clark County site (Table 1, Suppl. material 1)

#### Lasioglossum (Dialictus) hyalinum

Crawford, 1907

##### Notes

Collected from the Lewis and Clark County site (Table 1, Suppl. material 1)

#### Lasioglossum (Dialictus) marinense

(Michener, 1936)

##### Notes

Collected from the Lewis and Clark County, Park County and Flathead County sites (Table 1, Suppl. material 1)

#### Lasioglossum (Dialictus) nevadense

(Crawford, 1907)

##### Notes

Collected from the Lewis and Clark County site (Table 1, Suppl. material 1)

#### Lasioglossum (Dialictus) nigroviride

(Graenicher, 1911)

##### Notes

Collected from the Lewis and Clark County, Park County and Flathead County sites (Table 1, Suppl. material 1)

#### Lasioglossum (Dialictus) aff.lilliputense

Gibbs, 2010

##### Notes

Collected from the Lewis and Clark County site (Table 1, Suppl. material 1)

#### Lasioglossum (Dialictus) aff.occidentale

(Crawford, 1902)

##### Notes

Collected from the Lewis and Clark County site (Table 1, Suppl. material 1)

#### Lasioglossum (Dialictus) aff.pavoninum

(Ellis, 1913)

##### Notes

Collected from the Park County site (Table 1, Suppl. material 1)

#### Lasioglossum (Dialictus) obnubilum

(Sandhouse, 1924)

##### Notes

Collected from the Park County and Flathead County sites (Table 1, Suppl. material 1)

#### Lasioglossum (Dialictus) occidentale

(Crawford, 1902)

##### Notes

Collected from the Park County site (Table 1, Suppl. material 1)

#### Lasioglossum (Dialictus) planatum

(Lovell, 1905)

##### Notes

Collected from the Park County site (Table 1, Suppl. material 1)

#### Lasioglossum (Dialictus) pruinosum

(Robertson, 1892)

##### Notes

Collected from the Lewis and Clark County site (Table 1, Suppl. material 1)

#### Lasioglossum (Dialictus) ruidosense

(Cockerell, 1897)

##### Notes

Collected from the Lewis and Clark County and Park County sites (Table 1, Suppl. material 1)

#### Lasioglossum (Dialictus) sedi

(Sandhouse, 1924)

##### Notes

Collected from the Lewis and Clark County, Park County and Flathead county sites (Table 1, Suppl. material 1)

#### Lasioglossum (Dialictus) semicaeruleum

(Cockerell, 1895)

##### Notes

Collected from the Lewis and Clark County site (Table 1, Suppl. material 1)

#### Lasioglossum (Dialictus) sp. F17

##### Notes

Collected from the Park County site (Table 1, Suppl. material 1)

#### Lasioglossum (Dialictus) sp. F27

##### Notes

Collected from the Lewis and Clark County, Park County and Flathead county sites (Table 1, Suppl. material 1)

#### Lasioglossum (Dialictus) succinipenne

(Ellis, 1913)

##### Notes

Collected from the Lewis and Clark County site (Table 1, Suppl. material 1)

#### Lasioglossum (Dialictus) tenax

(Sandhouse, 1924)

##### Notes

Collected from the Park County site (Table 1, Suppl. material 1)

#### Lasioglossum (Dialictus) versatum

(Robertson, 1902)

##### Notes

Collected from the Flathead County site (Table 1, Suppl. material 1)

#### Lasioglossum (Dialictus) vierecki

(Crawford, 1904)

##### Notes

Collected from the Lewis and Clark County and Park County sites (Table 1, Suppl. material 1)

#### Lasioglossum (Evylaeus) sp. F1

##### Notes

Collected from the Lewis and Clark County, Park County and Flathead County sites (Table 1, Suppl. material 1)

#### Lasioglossum (Evylaeus) sp. F2

##### Notes

Collected from the Lewis and Clark County and Flathead County sites (Table 1, Suppl. material 1)

#### Lasioglossum (Evylaeus) sp. F3

##### Notes

Collected from the Lewis and Clark County and Park County sites (Table 1, Suppl. material 1)

#### Lasioglossum (Evylaeus) sp. F4

##### Notes

Collected from the Flathead County site (Table 1, Suppl. material 1)

#### Lasioglossum (Evylaeus) sp. F5

##### Notes

Collected from the Lewis and Clark County, Park County and Flathead County sites (Table 1, Suppl. material 1)

#### Lasioglossum (Evylaeus) sp. F6

##### Notes

Collected from the Park County and Flathead County sites (Table 1, Suppl. material 1)

#### Lasioglossum (Evylaeus) sp. F7

##### Notes

Collected from the Lewis and Clark County site (Table 1, Suppl. material 1)

#### Lasioglossum (Evylaeus) sp. F8

##### Notes

Collected from the Lewis and Clark County site (Table 1, Suppl. material 1)

#### Lasioglossum (Evylaeus) sp. F9

##### Notes

Collected from the Lewis and Clark County site (Table 1, Suppl. material 1)

#### Lasioglossum (Evylaeus) sp. F28

##### Notes

Collected from the Lewis and Clark County and Park County sites (Table 1, Suppl. material 1)

#### Lasioglossum (Lasioglossum) anhypops

McGinley, 1986

##### Notes

Collected from the Park County and Flathead County sites (Table 1, Suppl. material 1)

#### Lasioglossum (Lasioglossum) egregium

(Vachal, 1904)

##### Notes

Collected from the Lewis and Clark County and Park County sites (Table 1, Suppl. material 1)

#### Lasioglossum (Lasioglossum) paraforbesii

McGinley, 1986

##### Notes

Collected from the Lewis and Clark County site (Table 1, Suppl. material 1)

#### Lasioglossum (Lasioglossum) sisymbrii

(Cockerell, 1895)

##### Notes

Collected from the Lewis and Clark County and Park County sites (Table 1, Suppl. material 1)

#### Lasioglossum (Leuchalictus) leucozonium

(Schrank, 1781)

##### Notes

Collected from the Lewis and Clark County site (Table 1, Suppl. material 1)

#### Lasioglossum (Sphecodogastra) aberrans

(Crawford, 1903)

##### Notes

Collected from the Lewis and Clark County site (Table 1, Suppl. material 1)

#### Lasioglossum (Sphecodogastra) lusorium

(Cresson, 1872)

##### Notes

Collected from the Lewis and Clark County site (Table 1, Suppl. material 1)

#### Anthidiellum (Loyolanthidium) notatum Robertsoni

(Cockerell, 1904)

##### Notes

New species for Montana. Collected from the Lewis and Clark County site (Table 1, Suppl. material 1)

#### Anthidium (Anthidium) atrifrons

Cresson, 1868

##### Notes

Collected from the Lewis and Clark County and Park County sites (Table 1, Suppl. material 1)

#### Anthidium (Anthidium) clypeodentatum

Swenk, 1914

##### Notes

Collected from the Lewis and Clark County site (Table 1, Suppl. material 1)

#### Anthidium (Anthidium) formosum

Cresson, 1878

##### Notes

Collected from the Lewis and Clark County site (Table 1, Suppl. material 1)

#### Anthidium (Anthidium) mormonum

Cresson, 1878

##### Notes

Collected from the Lewis and Clark County and Park County sites (Table 1, Suppl. material 1)

#### Anthidium (Anthidium) placitum

Cresson, 1879

##### Notes

Collected from the Lewis and Clark County site (Table 1, Suppl. material 1)

#### Anthidium (Anthidium) tenuiflorae

Cockerell, 1907

##### Notes

Collected from the Lewis and Clark County site (Table 1, Suppl. material 1)

#### Anthidium (Anthidium) utahense

Swenk, 1914

##### Notes

Collected from the Lewis and Clark County site (Table 1, Suppl. material 1)

#### Ashmeadiella (Ashmeadiella) gillettei

Titus, 1904

##### Notes

Collected from the Lewis and Clark County site (Table 1, Suppl. material 1)

#### Ashmeadiella (Ashmeadiella) bucconis

(Say, 1837)

##### Notes

Collected from the Lewis and Clark County site (Table 1, Suppl. material 1)

#### Ashmeadiella (Ashmeadiella) cactorum

(Cockerell, 1897)

##### Notes

Collected from the Lewis and Clark County and Park County sites (Table 1, Suppl. material 1)

#### Ashmeadiella (Ashmeadiella) californica

(Ashmead, 1897)

##### Notes

Collected from the Lewis and Clark County and Park County sites (Table 1, Suppl. material 1)

#### Ashmeadiella (Ashmeadiella) meliloti

(Cockerell, 1897)

##### Notes

New species for Montana. Collected from the Lewis and Clark County site (Table 1, Suppl. material 1)

#### Ashmeadiella (Ashmeadiella) pronitens

(Cockerell, 1906)

##### Notes

New species for Montana. Collected from the Park County site (Table 1, Suppl. material 1)

#### Chelostoma (Chelostoma) minutum

Crawford, 1916

##### Notes

Collected from the Park County site (Table 1, Suppl. material 1)

#### Coelioxys (Boreocoelioxys) moesta

Cresson, 1864

##### Notes

Collected from the Lewis and Clark County, Park County and Flathead County sites (Table 1, Suppl. material 1)

#### Coelioxys (Boreocoelioxys) porterae

Cockerell, 1900

##### Notes

Collected from the Lewis and Clark County and Flathead County sites (Table 1, Suppl. material 1)

#### Coelioxys (Boreocoelioxys) rufitarsis

Smith, 1854

##### Notes

Collected from the Lewis and Clark County site (Table 1, Suppl. material 1)

#### Coelioxys (Coelioxys) sodalis

Cresson, 1878

##### Notes

Collected from the Lewis and Clark County site (Table 1, Suppl. material 1)

#### Coelioxys (Cyrtocoelioxys) modesta

Smith, 1854

##### Notes

Collected from the Lewis and Clark County, Park County and Flathead County sites (Table 1, Suppl. material 1)

#### Coelioxys (Paracoelioxys) funeraria

Smith, 1854

##### Notes

Collected from the Flathead County site (Table 1, Suppl. material 1)

#### Coelioxys (Synocoelioxys) alternata

Say, 1837

##### Notes

Collected from the Lewis and Clark County site (Table 1, Suppl. material 1)

#### Dianthidium (Dianthidium) cressonii

(Dalla Torre, 1896)

##### Notes

Collected from the Lewis and Clark County site (Table 1, Suppl. material 1)

#### Dianthidium (Dianthidium) subparvum

Swenk, 1914

##### Notes

Collected from the Lewis and Clark County site (Table 1, Suppl. material 1)

#### Dianthidium (Dianthidium) ulkei

(Cresson, 1878)

##### Notes

Collected from the Lewis and Clark County site (Table 1, Suppl. material 1)

#### Dioxys productus

(Cresson, 1879)

##### Notes

New species for Montana. Collected from the Lewis and Clark County site (Table 1, Suppl. material 1)

#### Heriades (Neotrypetes) carinatus

Cresson, 1864

##### Notes

Collected from the Lewis and Clark County and Flathead County sites (Table 1, Suppl. material 1)

#### Heriades (Neotrypetes) cressoni

Michener, 1938

##### Notes

Collected from the Lewis and Clark County and Park County sites (Table 1, Suppl. material 1)

#### Heriades (Neotrypetes) variolosa

(Cresson, 1872)

##### Notes

Collected from the Lewis and Clark County and Flathead County sites (Table 1, Suppl. material 1)

#### Hoplitis (Alcidamea) albifrons argentifrons

(Cresson, 1864)

##### Notes

Collected from the Park County site (Table 1, Suppl. material 1)

#### Hoplitis (Alcidamea) fulgida fulgida

(Cresson, 1864)

##### Notes

Collected from the Lewis and Clark County, Park County and Flathead County sites (Table 1, Suppl. material 1)

#### Hoplitis (Alcidamea) grinnelli

(Cockerell, 1910)

##### Notes

Collected from the Lewis and Clark County site (Table 1, Suppl. material 1)

#### Hoplitis (Alcidamea) hypocrita

(Cockerell, 1906)

##### Notes

Collected from the Lewis and Clark County site (Table 1, Suppl. material 1)

#### Hoplitis (Alcidamea) producta

(Cresson, 1864)

##### Notes

Collected from the Lewis and Clark County, Park County and Flathead County sites (Table 1, Suppl. material 1)

#### Hoplitis (Alcidamea) truncata

(Cresson, 1878)

##### Notes

Collected from the Lewis and Clark County site (Table 1, Suppl. material 1)

#### Hoplitis (Formicapis) robusta

(Nylander, 1848)

##### Notes

Collected from the Park County site (Table 1, Suppl. material 1)

#### Megachile (Argyropile) parallela

Smith, 1853

##### Notes

Collected from the Lewis and Clark County site (Table 1, Suppl. material 1)

#### Megachile (Chelostomoides) campanulae

(Robertson, 1903)

##### Notes

Collected from the Lewis and Clark County site (Table 1, Suppl. material 1)

#### Megachile (Chelostomoides) angelarum

Cockerell, 1902

##### Notes

Collected from the Lewis and Clark County site (Table 1, Suppl. material 1)

#### Megachile (Eutricharaea) apicalis

Spinola, 1808

##### Notes

Collected from the Lewis and Clark County site (Table 1, Suppl. material 1)

#### Megachile (Eutricharaea) rotundata

(Fabricius, 1793)

##### Notes

Collected from the Lewis and Clark County site (Table 1, Suppl. material 1)

#### Megachile (Litomegachile) brevis

Say, 1837

##### Notes

Collected from the Lewis and Clark County site (Table 1, Suppl. material 1)

#### Megachile (Litomegachile) onobrychidis

Cockerell, 1905

##### Notes

Collected from the Lewis and Clark County site (Table 1, Suppl. material 1)

#### Megachile (Litomegachile) texana

Cresson, 1878

##### Notes

Collected from the Lewis and Clark County site (Table 1, Suppl. material 1)

#### Megachile (Megachile) lapponica

Thomson, 1872

##### Notes

Collected from the Lewis and Clark County, Park County and Flathead County sites (Table 1, Suppl. material 1)

#### Megachile (Megachile) montivaga

Cresson, 1878

##### Notes

Collected from the Lewis and Clark County site (Table 1, Suppl. material 1)

#### Megachile (Megachile) relativa

Cresson, 1878

##### Notes

Collected from the Lewis and Clark County, Park County and Flathead County sites (Table 1, Suppl. material 1)

#### Megachile (Megachiloides) subnigra

Cresson, 1879

##### Notes

Collected from the Lewis and Clark County site (Table 1, Suppl. material 1)

#### Megachile (Megachiloides) wheeleri

Mitchell, 1927

##### Notes

Collected from the Lewis and Clark County site (Table 1, Suppl. material 1)

#### Megachile (Sayapis) fidelis

Cresson, 1878

##### Notes

Collected from the Lewis and Clark County site ((Table 1, Suppl. material 1)

#### Megachile (Sayapis) pugnata

Say, 1837

##### Notes

Collected from the Lewis and Clark County, Park County and Flathead County sites (Table 1, Suppl. material 1)

#### Megachile (Xanthosarus) frigida

Smith, 1853

##### Notes

Collected from the Lewis and Clark County, Park County and Flathead County sites (Table 1, Suppl. material 1)

#### Megachile (Xanthosarus) gemula

Cresson, 1878

##### Notes

Collected from the Lewis and Clark County, Park County and Flathead County sites (Table 1, Suppl. material 1)

#### Megachile (Xanthosarus) latimanus

Say, 1823

##### Notes

Collected from the Lewis and Clark County site (Table 1, Suppl. material 1)

#### Megachile (Xanthosarus) melanophaea

Smith, 1853

##### Notes

Collected from the Lewis and Clark County and Park County sites (Table 1, Suppl. material 1)

#### Megachile (Xanthosarus) perihirta

Cockerell, 1898

##### Notes

Collected from the Lewis and Clark County, Park County and Flathead County sites (Table 1, Suppl. material 1)

#### Osmia (Cephalosmia) californica

Cresson, 1864

##### Notes

Collected from the Lewis and Clark County site (Table 1, Suppl. material 1)

#### Osmia (Cephalosmia) marginipennis

Cresson, 1878

##### Notes

Collected from the Lewis and Clark County site (Table 1, Suppl. material 1)

#### Osmia (Cephalosmia) montana montana

Cresson, 1864

##### Notes

Collected from the Lewis and Clark County and Park County sites (Table 1, Suppl. material 1)

#### Osmia (Cephalosmia) subaustralis

Cockerell, 1900

##### Notes

Collected from the Park County site (Table 1, Suppl. material 1)

#### Osmia (Hapsidosmia) iridis

Cockerell and Titus, 1902

##### Notes

Collected from the Lewis and Clark County site (Table 1, Suppl. material 1)

#### Osmia (Helicosmia) coloradensis

Cresson, 1878

##### Notes

Collected from the Lewis and Clark County, Park County and Flathead County sites (Table 1, Suppl. material 1)

#### Osmia (Helicosmia) texana

Cresson, 1872

##### Notes

Collected from the Lewis and Clark County site (Table 1, Suppl. material 1)

#### Osmia (Melanosmia) aff.albolateralis

Cockerell, 1906

##### Notes

Collected from the Lewis and Clark County site (Table 1, Suppl. material 1)

#### Osmia (Melanosmia) aff.grindeliae

Cockerell, 1900

##### Notes

Collected from the Lewis and Clark County site (Table 1, Suppl. material 1)

#### Osmia (Melanosmia) aff.paradisica

Sandhouse, 1924

##### Notes

Collected from the Lewis and Clark County, Park County and Flathead County sites (Table 1, Suppl. material 1)

#### Osmia (Melanosmia) aff.pusilla

Cresson, 1864

##### Notes

Collected from the Park County site (Table 1, Suppl. material 1)

#### Osmia (Melanosmia) albolateralis

Cockerell, 1906

##### Notes

Collected from the Lewis and Clark County, Park County and Flathead County sites (Table 1, Suppl. material 1)

#### Osmia (Melanosmia) atrocyanea

Cockerell, 1897

##### Notes

Collected from the Lewis and Clark County site (Table 1, Suppl. material 1)

#### Osmia (Melanosmia) brevis

Cresson, 1864

##### Notes

Collected from the Lewis and Clark County and Park County sites (Table 1, Suppl. material 1)

#### Osmia (Melanosmia) bruneri

Cockerell, 1897

##### Notes

Collected from the Lewis and Clark County site (Table 1, Suppl. material 1)

#### Osmia (Melanosmia) bucephala

Cresson, 1864

##### Notes

Collected from the Lewis and Clark County, Park County and Flathead County sites (Table 1, Suppl. material 1)

#### Osmia (Melanosmia) cyanella

Cockerell, 189

##### Notes

Collected from the Flathead County site (Table 1, Suppl. material 1)

#### Osmia (Melanosmia) densa

Cresson, 1864

##### Notes

Collected from the Lewis and Clark County, Park County and Flathead County sites (Table 1, Suppl. material 1)

#### Osmia (Melanosmia) ednae

Cockerell, 1907

##### Notes

Collected from the Park County site (Table 1, Suppl. material 1)

#### Osmia (Melanosmia) grindeliae

Cockerell, 1910

##### Notes

Collected from the Lewis and Clark County, Park County and Flathead County sites (Table 1, Suppl. material 1)

#### Osmia (Melanosmia) inermis

(Zetterstedt, 1838)

##### Notes

Collected from the Lewis and Clark County, Park County and Flathead County sites (Table 1, Suppl. material 1)

#### Osmia (Melanosmia) integra

Cresson, 1878

##### Notes

Collected from the Lewis and Clark County site (Table 1, Suppl. material 1)

#### Osmia (Melanosmia) juxta

Cresson, 1864

##### Notes

Collected from the Lewis and Clark County, Park County and Flathead County sites (Table 1, Suppl. material 1)

#### Osmia (Melanosmia) kincaidii

Cockerell, 1897

##### Notes

Collected from the Lewis and Clark County site (Table 1, Suppl. material 1)

#### Osmia (Melanosmia) longula

Cresson, 1864

##### Notes

Collected from the Lewis and Clark County and Park County sites (Table 1, Suppl. material 1)

#### Osmia (Melanosmia) malina

Cockerell, 1909

##### Notes

Collected from the Lewis and Clark County site (Table 1, Suppl. material 1)

#### Osmia (Melanosmia) nigrifrons

Cresson, 1878

##### Notes

Collected from the Lewis and Clark County and Park County sites (Table 1, Suppl. material 1)

#### Osmia (Melanosmia) nigriventris

(Zetterstedt, 1838)

##### Notes

Collected from the Park County site (Table 1, Suppl. material 1)

#### Osmia (Melanosmia) odontogaster gr.sp.1

##### Notes

Collected from the Lewis and Clark County and Park County sites (Table 1, Suppl. material 1)

#### Osmia (Melanosmia) odontogaster gr.sp.2

##### Notes

Collected from the Park County site (Table 1, Suppl. material 1)

#### Osmia (Melanosmia) paradisica

Sandhouse, 1924

##### Notes

Collected from the Park County site (Table 1, Suppl. material 1)

#### Osmia (Melanosmia) pentstemonis

Cockerell, 1906

##### Notes

Collected from the Lewis and Clark County, Park County and Flathead County sites (Table 1, Suppl. material 1)

#### Osmia (Melanosmia) phaceliae

Cockerell, 1907

##### Notes

Collected from the Lewis and Clark County, Park County and Flathead County sites (Table 1, Suppl. material 1)

#### Osmia (Melanosmia) physariae

Cockerell, 1907

##### Notes

Collected from the Park County site (Table 1, Suppl. material 1)

#### Osmia (Melanosmia) pikei

Cockerell, 1907

##### Notes

Collected from the Park County site (Table 1, Suppl. material 1)

#### Osmia (Melanosmia) proxima

Cresson, 1864

##### Notes

Collected from the Park County site (Table 1, Suppl. material 1)

#### Osmia (Melanosmia) pusilla

Cresson, 1864

##### Notes

Collected from the Lewis and Clark County, Park County and Flathead County sites (Table 1, Suppl. material 1)

#### Osmia (Melanosmia) sculleni

Sandhouse, 1939

##### Notes

Collected from the Park County site (Table 1, Suppl. material 1)

#### Osmia (Melanosmia) simillima

Smith, 1853

##### Notes

Collected from the Lewis and Clark County site (Table 1, Suppl. material 1)

#### Osmia (Melanosmia) sp.3

##### Notes

Collected from the Flathead County site (Table 1, Suppl. material 1)

#### Osmia (Melanosmia) sp.9

##### Notes

Collected from the Lewis and Clark County site (Table 1, Suppl. material 1)

#### Osmia (Melanosmia) tersula

Cockerell, 1912

##### Notes

Collected from the Park County site (Table 1, Suppl. material 1)

#### Osmia (Melanosmia) trevoris

Cockerell, 1897

##### Notes

Collected from the Lewis and Clark County site (Table 1, Suppl. material 1)

#### Osmia (Melanosmia) tristella

Cockerell, 1897

##### Notes

Collected from the Lewis and Clark County, Park County and Flathead County sites (Table 1, Suppl. material 1)

#### Osmia (Osmia) lignaria propinqua

Cresson, 1864

##### Notes

Collected from the Lewis and Clark County and Park County sites (Table 1, Suppl. material 1)

#### Stelis (Stelis) aff. permaculata

Cockerell, 1898

##### Notes

Collected from the Lewis and Clark County site (Table 1, Suppl. material 1)

#### Stelis (Stelis) calliphorina

(Cockerell, 1911)

##### Notes

Collected from the Lewis and Clark County site (Table 1, Suppl. material 1)

#### Stelis (Stelis) callura

Cockerell, 1925

##### Notes

Collected from the Lewis and Clark County site (Table 1, Suppl. material 1)

#### Stelis (Stelis) carnifex

Cockerell, 1911

##### Notes

Collected from the Lewis and Clark County and Park County sites (Table 1, Suppl. material 1)

#### Stelis (Stelis) foederalis gr.sp.2

##### Notes

Collected from the Lewis and Clark County site (Table 1, Suppl. material 1)

#### Stelis (Stelis) foederalis gr.sp.6

##### Notes

Collected from the Lewis and Clark County and Flathead County sites (Table 1, Suppl. material 1)

#### Stelis (Stelis) foederalis gr.sp.7

##### Notes

Collected from the Park County site (Table 1, Suppl. material 1)

#### Stelis (Stelis) foederalis gr.sp.8

##### Notes

Collected from the Park County site (Table 1, Suppl. material 1)

#### Stelis (Stelis) montana

Cresson, 1864

##### Notes

Collected from the Lewis and Clark County, Park County and Flathead County sites (Table 1, Suppl. material 1)

#### Stelis (Stelis) monticola

Cresson, 1878

##### Notes

Collected from the Lewis and Clark County and Park County sites (Table 1, Suppl. material 1)

#### Stelis (Stelis) nitida

Cresson, 1878

##### Notes

Collected from the Park County site (Table 1, Suppl. material 1)

#### Stelis (Stelis) permaculata

Cockerell, 1898

##### Notes

Collected from the Lewis and Clark County site (Table 1, Suppl. material 1)

#### Anthophora (Clisodon) terminalis

Cresson, 1869

##### Notes

Collected from the Lewis and Clark County and Park County sites (Table 1, Suppl. material 1)

#### Anthophora (Lophanthophora) pacifica

Cresson, 1878

##### Notes

Collected from the Lewis and Clark County site (Table 1, Suppl. material 1)

#### Anthophora (Lophanthophora) ursina

Cresson, 1869

##### Notes

Collected from the Lewis and Clark County site (Table 1, Suppl. material 1)

#### Anthophora (Melea) bomboides

Kirby, 1838

##### Notes

Collected from the Lewis and Clark County site (Table 1, Suppl. material 1)

#### Anthophora (Mystacanthophora) urbana

Cresson, 1878

##### Notes

Collected from the Lewis and Clark County site (Table 1, Suppl. material 1)

#### Apis mellifera

Linnaeus, 1758

##### Notes

Collected from the Lewis and Clark County and Park County sites (Table 1, Suppl. material 1)

#### Bombus (Bombus) occidentalis

Greene, 1858

##### Notes

Collected from the Park County and Flathead County sites (Table 1, Suppl. material 1)

#### Bombus (Cullumanobombus) griseocollis

(De Geer, 1773)

##### Notes

Collected from the Lewis and Clark County site (Table 1, Suppl. material 1)

#### Bombus (Cullumanobombus) rufocinctus

Cresson, 1863

##### Notes

Collected from the Lewis and Clark County, Park County and Flathead County sites (Table 1, Suppl. material 1)

#### Bombus (Psithyrus) flavidus

Eversmann, 1852

##### Notes

Collected from the Park County and Flathead County sites (Table 1, Suppl. material 1)

#### Bombus (Psithyrus) insularis

(Smith, 1861)

##### Notes

Collected from the Lewis and Clark County, Park County and Flathead County sites (Table 1, Suppl. material 1)

#### Bombus (Psithyrus) suckleyi

Greene, 1860

##### Notes

Collected from the Flathead County site (Table 1, Suppl. material 1)

#### Bombus (Pyrobombus) bifarius

Cresson, 1878

##### Notes

Collected from the Lewis and Clark County, Park County and Flathead County sites (Table 1, Suppl. material 1)

#### Bombus (Pyrobombus) centralis

Cresson, 1864

##### Notes

Collected from the Lewis and Clark County, Park County and Flathead County sites (Table 1, Suppl. material 1)

#### Bombus (Pyrobombus) flavifrons

Cresson, 1863

##### Notes

Collected from the Lewis and Clark County, Park County and Flathead County sites (Table 1, Suppl. material 1)

#### Bombus (Pyrobombus) huntii

Greene, 1860

##### Notes

Collected from the Lewis and Clark County and Park County sites (Table 1, Suppl. material 1)

#### Bombus (Pyrobombus) melanopygus

Nylander, 1848

##### Notes

Collected from the Park County and Flathead County sites (Table 1, Suppl. material 1)

#### Bombus (Pyrobombus) mixtus

Cresson, 1878

##### Notes

Collected from the Park County and Flathead County sites (Table 1, Suppl. material 1)

#### Bombus (Pyrobombus) sitkensis

Nylander, 1848

##### Notes

Collected from the Flathead County site (Table 1, Suppl. material 1)

#### Bombus (Pyrobombus) vagans

Smith, 1854

##### Notes

Collected from the Flathead County site (Table 1, Suppl. material 1)

#### Bombus (Subterraneobombus) appositus

Cresson, 1878

##### Notes

Collected from the Lewis and Clark County, Park County and Flathead County sites (Table 1, Suppl. material 1)

#### Bombus (Subterraneobombus) borealis

Kirby, 1837

##### Notes

Collected from the Lewis and Clark County site (Table 1, Suppl. material 1)

#### Bombus (Thoracobombus) californicus

Smith, 1854

##### Notes

Collected from the Flathead County site (Table 1, Suppl. material 1)

#### Bombus (Thoracobombus) fervidus

(Fabricius, 1798)

##### Notes

Collected from the Lewis and Clark County and Park County sites (Table 1, Suppl. material 1)

#### Ceratina (Zadontomerus) nanula

Cockerell, 1897

##### Notes

Collected from the Lewis and Clark County, Park County and Flathead County sites (Table 1, Suppl. material 1)

#### Ceratina (Zadontomerus) neomexicana

Cockerell, 1901

##### Notes

Collected from the Lewis and Clark County site (Table 1, Suppl. material 1)

#### Diadasia (Coquillettapis) diminuta

(Cresson, 1878)

##### Notes

Collected from the Lewis and Clark County site (Table 1, Suppl. material 1)

#### Epeolus sp.

##### Notes

Collected from the Lewis and Clark County site (Table 1, Suppl. material 1)

#### Eucera (Synhalonia) edwardsii

(Cresson, 1878)

##### Notes

Collected from the Lewis and Clark County site (Table 1, Suppl. material 1)

#### Eucera (Synhalonia) frater

(Cresson, 1878)

##### Notes

Collected from the Lewis and Clark County site (Table 1, Suppl. material 1)

#### Eucera (Synhalonia) fulvitarsis

(Cresson, 1878)

##### Notes

Collected from the Lewis and Clark County site (Table 1, Suppl. material 1)

#### Melecta (Melecta) pacifica fulvida

Cresson, 1878

##### Notes

Collected from the Lewis and Clark County site (Table 1, Suppl. material 1)

#### Melecta (Melecta) separata

Cresson, 1879

##### Notes

Collected from the Lewis and Clark County site (Table 1, Suppl. material 1)

#### Melissodes (Eumelissodes) confusa

Cresson, 1878

##### Notes

Collected from the Lewis and Clark County site (Table 1, Suppl. material 1)

#### Melissodes (Eumelissodes) coreopsis

Robertson, 1905

##### Notes

Collected from the Lewis and Clark County site (Table 1, Suppl. material 1)

#### Melissodes (Eumelissodes) hymenoxidis

Cockerell, 1906

##### Notes

Collected from the Lewis and Clark County site (Table 1, Suppl. material 1)

#### Melissodes (Eumelissodes) microstictus

Cockerell, 1905

##### Notes

Collected from the Lewis and Clark County and Park County sites (Table 1, Suppl. material 1)

#### Melissodes (Eumelissodes) utahensis

LaBerge, 1961

##### Notes

Collected from the Lewis and Clark County site (Table 1, Suppl. material 1)

#### Melissodes (Heliomelissodes) rivalis

Cresson, 1872

##### Notes

Collected from the Lewis and Clark County site (Table 1, Suppl. material 1)

#### Melissodes sp. F1

##### Notes

Collected from the Lewis and Clark County site (Table 1, Suppl. material 1)

#### Melissodes sp. F2

##### Notes

Collected from the Lewis and Clark County site (Table 1, Suppl. material 1)

#### Melissodes unk1

##### Notes

Collected from the Lewis and Clark County site (Table 1, Suppl. material 1)

#### Nomada edwardsii

Cresson, 1878

##### Notes

Collected from the Lewis and Clark County site (Table 1, Suppl. material 1)

#### Nomada sp. F1

##### Notes

Collected from the Lewis and Clark County site (Table 1, Suppl. material 1)

#### Nomada sp. F2

##### Notes

Collected from the Lewis and Clark County site (Table 1, Suppl. material 1)

#### Nomada sp. F3

##### Notes

Collected from the Lewis and Clark County and Park County sites (Table 1, Suppl. material 1)

#### Nomada sp. F4

##### Notes

Collected from the Lewis and Clark County site (Table 1, Suppl. material 1)

#### Nomada sp. F5

##### Notes

Collected from the Lewis and Clark County site (Table 1, Suppl. material 1)

#### Nomada sp. F6

##### Notes

Collected from the Park County site (Table 1, Suppl. material 1)

#### Nomada sp. F7

##### Notes

Collected from the Lewis and Clark County site (Table 1, Suppl. material 1)

#### Nomada sp. F8

##### Notes

Collected from the Flathead County site (Table 1, Suppl. material 1)

#### Triepeolus paenepectoralis

Viereck, 1905

##### Notes

Collected from the Lewis and Clark County site (Table 1, Suppl. material 1)

## Analysis

We collected 8011 bee specimens representing 281 species and morphospecies in 32 genera and five families between 2013 and 2016. Following are the total number of specimens by family (to avoid inflating species richness, we did not include male morphospecies in species counts because female morphospecies may have been the same species): Colletidae: 357 specimens, 2 genera, 17 species; Andrenidae: 395 specimens, 3 genera, 44 species and morphospecies; Halictidae: 1251 specimens, 4 genera, 55 species and morphospecies; Megachilidae: 2920 specimens, 12 genera, 113 species and morphospecies; Apidae: 3088 specimens, 11 genera, 52 species and morphospecies.

Though resources did not allow for a comprehensive literature search to assess new state records for all the species on this checklist, an exploratory search for 25 of the species (in no particular order) revealed no previously published records in Montana for *Andrena saccata* Viereck; *Anthidiellum notatum robertsoni* (Cockerell); *Ashmeadiella meliloti* (Cockerell); *Ashmeadiella pronitens* (Cockerell); *Colletes lutzi lutzi* Timberlake; and *Dioxys productus* (Cresson). In addition, Dolan et al. (2017) reported a specimen of *Bombus borealis* Kirby from this study to be a first Montana state record. In effect, this brings the number of known bee species in Montana up from 366 (Kuhlman and Burrows 2017) to at least 372, and indicates a clear need for more extensive bee surveys in the state.

## Discussion

Because Montana spans a large area of diverse topography, landscape and climate (Dolan et al. 2017), species groups are likely to vary among different locations. For instance, compared with a checklist of bees from a similar montane habitat in Montana (Kuhlman and Burrows 2017), there was some overlap among species, with 121 species (not including morphospecies) in common with our list, but also some variability, with 126 species unique to our study. When compared to an unpublished study on bees associated with native flower strips around agricultural fields in the Gallatin Valley, Montana (C.M. Delphia pers. com.), the species suites included 117 species in common and 130 unique to our study. We expect that future biosurvey efforts will reveal hundreds of species not yet listed in Montana.

There is growing evidence of declines in wild bee species diversity (Winfree et al. 2009, Winfree 2010, Potts et al. 2010, Burkle et al. 2013, Goulson et al. 2015, Woodcock et al. 2016, Koh et al. 2015). In order to determine whether bee declines are occurring, baseline data or repeated monitoring surveys are required. The lack of comprehensive surveys or monitoring of native bee populations across Montana has resulted in a lack of species lists for the state as well as difficulty in tracking population, species, and community trends. This checklist adds to the basic understanding of native bee fauna, and we hope it will stimulate further research in this important field.

## Supplementary Material

Supplementary material 1 Supplementary Table1 Data type: Species Collection Dates
File: oo_236872.pdf Elizabeth G. Reese, Laura A. Burkle, Casey M. Delphia, Terry Griswold

## Figures and Tables

**Table:** 

Flathead Co. (Kalispell)	Lewis & Clark Co. (Helena)	Park Co. (Livingston)
coordinates (latitude, longitude)	48.6, -114.3	46.7, -111.7	45.2, -110.4
temperature (°C)	-2.2	15.8	-13
precipitation (mm)	684	351	632
elevation (m)	1312	1373	2248

**Table 2.** List of published keys used for species identification.

**Family**	**Genus**	**Reference**
Andrenidae	* Andrena *	Bouseman and LaBerge 1978, Donovan 1977, LaBerge 1969, LaBerge and Ribble 1972,LaBerge 1973, LaBerge 1977, LaBerge 1980, LaBerge 1985, LaBerge 1986, LaBerge and Ribble 1975, Ribble 1974, Ribble 1968, Thorp 1969
	* Panurginus *	unpublished works
	* Protandrena *	Timberlake 1976
Apidae	* Anthophora *	Ascher and Pickering 2016
	* Bombus *	Koch et al. 2012, Thorp et al. 1983, Williams et al. 2014
	* Ceratina *	Daly 1973
	* Diadasia *	Sipes 2001
	* Epeolus *	Brumley 1965
	* Eucera *	Timberlake 1969
	* Melecta *	Hurd and Linsley 1951
	* Melissodes *	LaBerge 1956a, LaBerge 1956b, LaBerge 1961
	* Triepeolus *	Rightmyer 2008
Colletidae	* Colletes *	Stephen 1954
	* Hylaeus *	Snelling 1966a, Snelling 1966b, Snelling 1970
Halictidae	* Agapostemon *	Roberts 1973b
	* Dufourea *	Dumesh and Sheffield 2012
	* Halictus *	Roberts 1973a
	* Lasioglossum *	Gibbs 2010, McGinley 1986, McGinley 2003
Megachilidae	* Anthidiellum *	Hurd and Michener 1955
	* Anthidium *	Gonzalez and Griswold 2013
	* Ashmeadiella *	Hurd and Michener 1955
	* Chelostoma *	Michener 1938
	* Coelioxys *	Baker 1975, DeSilva 2012
	* Dianthidium *	Grigarick and Stange 1968
	* Dioxys *	Hurd 1958
	* Heriades *	Hurd and Michener 1955
	* Hoplitis *	Michener 1947
	* Megachile *	Sheffield et al. 2011
	* Osmia *	Sandhouse 1939, Rightmyer et al. 2010
	* Stelis *	unpublished works
