# Supplementary material for: A list of bees from three locations in the Northern Rockies Ecoregion (NRE) of western Montana
Source: Biodivers Data J. 2018 Oct 30;(6):e27161. doi: 10.3897/BDJ.6.e27161 (PMC6220116; doi:10.3897/BDJ.6.e27161)
Supplement: Supplementary material 1 — Supplementary Table1 [file bdj-06-e27161-s001.pdf]

Species	Authorship	Dates Collected Park Co.	Dates Collected Lewis and Clark Co.	Dates Collected Flathead Co.
Colletidae				
Colletes consors consors	Cresson, 1868		June 2014<br>June 2015	
Colletes fulgidus	Swenk, 1904	July-August 2014<br>July 2015<br>July-August 2016	July 2013<br>June-August 2014<br>June-August 2015<br>July-August 2016	
Colletes hyalinus hyalinus	Provancher, 1888	July-August 2014<br>August 2016		
Colletes kincaidii	Cockerell, 1898	August 2016	July 2013<br>June-August 2014<br>July 2016	
Colletes lutzi lutzi	Timberlake, 1943	July 2014<br>July 2016	July-August 2015	
Colletes phaceliae	Cockerell, 1906		June-July 2013<br>July-August 2014<br>June-July 2015<br>June-July 2016	
Hylaeus (Cephalylaeus) basalis	(Smith, 1853)	July-August 2014<br>June-July 2015<br>June-July 2016		July 2014
Hylaeus (Hylaeus) annulatus	(Linnaeus, 1758)	June 2013<br>July-August 2014	June 2013<br>July-August 2014	July-August 2014
Hylaeus (Hylaeus) leptocephalus	(Morawitz, 1871)		July-August 2014<br>July 2015<br>August 2016	
Hylaeus (Hylaeus) mesillae	(Cockerell, 1896)		June 2013<br>June 2014	
Hylaeus (Hylaeus) rudbeckiae	(Cockerell and Casad, 1895)		June 2013<br>June-August 2014<br>June-July 2015<br>June 2106	

Species	Authorship	Dates Collected Park Co.	Dates Collected Lewis and Clark Co.	Dates Collected Flathead Co.
Hylaeus (Hylaeus) verticalis	(Cresson, 1869)	July-August 2014	June 2013<br>July 2014<br>June 2015<br>June 2016	June-July 2014
Hylaeus (Paraprosopis) coloradensis	(Cockerell, 1896)	July-August 2014	July-August 2014<br>June-August 2015<br>July-August 2016	
Hylaeus (Paraprosopis) nevadensis	(Cockerell, 1896)		July 2015	
Hylaeus (Paraprosopis) wootoni	(Cockerell, 1896)	July-August 2014<br>June-August 2016	July-August 2014<br>June-July 2015<br>August 2016	
Hylaeus (Prosopis) episcopalis	(Cockerell, 1896)	August 2014<br>August 2015	July 2014	
Hylaeus (Prosopis) modestus	Say, 1837	July-August 2014<br>July-August 2015<br>June-August 2016	July 2014<br>June 2016	July 2013<br>July 2014
Andrenidae				
Andrena (Andrena) milwaukeensis	Graenicher, 1903	July 2015	June 2015	
Andrena (Andrena) saccata	Viereck, 1904	July 2013<br>July 2014<br>June-July 2015<br>June 2016		
Andrena (Andrena) thaspii	Graenicher, 1903	July-August 2014<br>July 2015	June 2013<br>June 2014<br>June 2015	July 2014
Andrena (Andrena) topazana	Cockerell, 1906	June-July 2014<br>June 2015<br>June 2016		
*Andrena (Andrena) sp.*F1			June 2016	
Andrena (Cnemidandrena) surda	Cockerell, 1910	July 2014		
Andrena (Diandrena) evoluta	Linsley & MacSwain, 1961		June 2015	

Species	Authorship	Dates Collected Park Co.	Dates Collected Lewis and Clark Co.	Dates Collected Flathead Co.
Andrena (Euandrena) lawrencei	Viereck, & Cockerell, 1914		June 2013	
			May-June 2014	
			June 2015	
			May-June 2016	
Andrena (Euandrena) nigrocaerulea	Cockerell, 1897	June 2016	June 2014	
Andrena (Geissandrena) trevoris	Cockerell, 1897		June 2015	
Andrena (Melandrena) nivalis	Smith, 1853		June 2013	July 2014
			June-July 2014	
			June 2015	
			July 2016	
Andrena (Melandrena) pertristis	Cockerell, 1905		June 2015	
Andrena (Melandrena) transnigra	Viereck, 1904		May 2014	
Andrena (Melandrena) vicina	Smith, 1853		June 2015	
Andrena (Micrandrena) melanochroa	Cockerell, 1898	July 2015	June 2015	
Andrena (Micrandrena) microchlora	Cockerell, 1922		May 2014	
Andrena (Plastandrena) crataegi	Robertson, 1893	July 2014	June 2013	
		June-July 2015	May-June 2014	
			June 2015	
			May-June 2016	
Andrena (Plastandrena) prunorum	Cockerell, 1896	June 2015	June 2013	
			June-July 2014	
			June 2015	
			June-July 2016	
Andrena (Scaphandrena) aff. shoshoni	Ribble, 1974		May 2014	
Andrena (Scaphandrena) scurra	Viereck, 1904		June 2015	
			June 2016	
Andrena (Scaphandrena) walleyi	Cockerell, 1932		May 2014	
			May-June 2016	
Andrena (Thysandrena) candida	Smith, 1879	July 2014		June 2013
Andrena (Thysandrena) knuthiana	Cockerell, 1901	July 2014		July 2014
Andrena (Thysandrena) medionitens	Cockerell, 1902	June 2016	July 2014	
			June 2015	
			June 2016	

Species	Authorship	Dates Collected Park Co.	Dates Collected Lewis and Clark Co.	Dates Collected Flathead Co.
Andrena (Thysandrena) vierecki	Cockerell, 1904	July 2014		
Andrena (Trachandrena) amphibola	(Viereck, 1904)	June-July 2013<br>July 2014<br>June 2015	June 2013<br>June-August 2014<br>June-July 2015<br>June-July 2016	
Andrena (Trachandrena) cleodora	(Viereck, 1904)		July 2014	June-July 2013<br>July 2014
Andrena (Trachandrena) cupreotincta	Cockerell, 1901		June 2015	
Andrena (Trachandrena) miranda	Smith, 1879	July 2013<br>July-August 2014<br>June-July 2015<br>June-August 2016	June-July 2014<br>June 2016	
Andrena (Trachandrena) salicifloris	Cockerell, 1897	June 2015	July 2014	
Andrena (Trachandrena) sigmundi	Cockerell, 1902		June 2015	
*Andrena sp.*F7		July 2015	June 2015	
*Andrena sp.*F8			May 2016	
*Andrena sp.*F11			June 2016	
*Andrena sp.*F12			May 2016	
*Andrena sp.*F13			June 2016	
*Andrena sp.*F14			May 2016	
*Andrena sp.*F15		June 2016		
Andrena sp.F16			June 2016	
*Andrena sp.*F17			June 2016	
Panurginus atriceps	(Cresson, 1878)	July 2014	June 2013<br>June 2014<br>June 2015	June-July 2014
*Panurginus sp.*F1		June 2015		
*Panurginus sp.*1		July 2014<br>June 1015	June 2015	
Protandrena (Pterosarus) innuptus	(Cockerell, 1896)		July 2013	

Species	Authorship	Dates Collected Park Co.	Dates Collected Lewis and Clark Co.	Dates Collected Flathead Co.
Halictidae				
Agapostemon (Agapostemon) texanus	Cresson, 1872		July 2013<br>May-August 2014<br>June 2015<br>June-August 2016	
Agapostemon (Agapostemon) virescens	(Fabricius, 1775)		June 2013<br>June-August 2014<br>June-July 2015<br>June-July 2016	
Dufourea dilatipes	Bohart, 1948			June 2015
Dufourea maura	(Cresson, 1878)	July 2013<br>July-August 2014<br>June-July 2015<br>July 2016	June 2013<br>June-July 2014<br>June 2015<br>June 2016	July 2013
Dufourea trochantera	Bohart, 1948		June 2013<br>June-July 2014<br>June 2015	
Halictus (Nealictus) farinosus	Smith, 1853	August 2016	August 2014<br>August 2016	
Halictus (Odontalictus) ligatus	Say, 1837		June-July 2013<br>May-August 2014<br>June-August 2015<br>June-August 2016	
Halictus (Protohalictus) rubicundus	(Christ, 1791)	June-August 2014<br>June-July 2015<br>June 2016	June 2013<br>June-August 2014<br>June-July 2015<br>July 2016	June 2013<br>June-August 2014<br>June-July 2015
Halictus (Seladonia) confusus	Smith, 1853	July-August 2014<br>June 2015<br>June 2016	June 2013<br>May-August 2014<br>June-August 2015<br>June-July 2016	July 2014

Species	Authorship	Dates Collected Park Co.	Dates Collected Lewis and Clark Co.	Dates Collected Flathead Co.
Halictus (Seladonia) tripartitus	Cockerell, 1895		June 2013<br>May-August 2014<br>June-August 2015<br>June-July 2016	
Lasioglossum (Dialictus) abundipunctum	Gibbs, 2010		July 2014<br>June 2015	
Lasioglossum (Dialictus) aff.*caducum*	(Sandhouse, 1924)		May-August 2014<br>June-August 2015	
Lasioglossum (Dialictus) aff.*nevadense*	(Crawford, 1907)		July 2014<br>June 2015<br>August 2016	
Lasioglossum (Dialictus) albipenne	(Robertson, 1890)	July-August 2014<br>June 2016	June 2013<br>May-August 2014<br>June-July 2015<br>June-August 2016	
Lasioglossum (Dialictus) brunneiventre	(Crawford, 1907)		July 2016	
Lasioglossum (Dialictus) ebmerellum	Gibbs, 2010		July 2013<br>June-August 2014<br>June-August 2015<br>June-July 2016	
Lasioglossum (Dialictus) ephialtum	Gibbs, 2010	June 2015		July 2013<br>July 2014
Lasioglossum (Dialictus) hudsoniellum	(Cockerell, 1919)		July 2014	
Lasioglossum (Dialictus) hyalinum	Crawford, 1907		July 2014<br>June 2015	
Lasioglossum (Dialictus) marinense	(Michener, 1936)	June 2013<br>June-August 2014<br>June 2015<br>July 2016	May-July 2014<br>June-July 2015<br>June July 2016	June-July 2013<br>June-August 2014<br>June-July 2015
Lasioglossum (Dialictus) nevadense	(Crawford, 1907)		July-August 2016	

Species	Authorship	Dates Collected Park Co.	Dates Collected Lewis and Clark Co.	Dates Collected Flathead Co.
Lasioglossum (Dialictus) nigroviride	(Graenicher, 1911)	June 2013<br>June-August 2014<br>June 2015<br>August 2016	June 2015	July 2013<br>June-August 2014<br>July 2015
Lasioglossum (Dialictus) aff.*lilliputense*	Gibbs, 2010		July 2015	
Lasioglossum (Dialictus) aff.*occidentale*	(Crawford, 1902)		June-July 2013<br>June-August 2014<br>June-July 2015	
Lasioglossum (Dialictus) aff.*pavoninum*	(Ellis, 1913)		June 2013<br>June-July 2014	
Lasioglossum (Dialictus) obnubilum	(Sandhouse, 1924)	August 2014	June-July 2014<br>June 2015	
Lasioglossum (Dialictus) occidentale	(Crawford, 1902)		June-July 2013<br>June-August 2014<br>June-July 2015	
Lasioglossum (Dialictus) planatum	(Lovell, 1905)	June 2016		
Lasioglossum (Dialictus) pruinosum	(Robertson, 1892)		May-August 2014<br>June-July 2015<br>July 2016	
Lasioglossum (Dialictus) ruidosense	(Cockerell, 1897)	July 2014	May-June 2014<br>June 2015<br>June 2016	
Lasioglossum (Dialictus) sedi	(Sandhouse, 1924)	July 2014	June 2013<br>June-July 2014<br>June-July 2015<br>June-July 2016	
Lasioglossum (Dialictus) semicaeruleum	(Cockerell, 1895)		May-July 2014<br>June 2016	
*Lasioglossum (Dialictus) sp.*F17		June-July 2014		
*Lasioglossum (Dialictus) sp.*F27		July 2014	July 2015	June 2015
Lasioglossum (Dialictus) succinipenne	(Ellis, 1913)		May-August 2014<br>June-July 2015<br>June-August 2016	

Species	Authorship	Dates Collected Park Co.	Dates Collected Lewis and Clark Co.	Dates Collected Flathead Co.
Lasioglossum (Dialictus) tenax	(Sandhouse, 1924)	June-August 2014<br>June-July 2015	May-August 2014<br>June-July 2015	
Lasioglossum (Dialictus) versatum	(Robertson, 1902)			June 2015
Lasioglossum (Dialictus) vierecki	(Crawford, 1904)		August 2014	
*Lasioglossum (Evylaeus) sp.*F1		June-July 2014<br>June 2015	July 2015	June-July 2014
*Lasioglossum (Evylaeus) sp.*F2		August 2016	May-August 2014<br>June 2015<br>May-July 2016	June 2013<br>July 2015
*Lasioglossum (Evylaeus) sp.*F3		June 2013<br>June-August 2014<br>July 2015	June 2015	
*Lasioglossum (Evylaeus) sp.*F4				July 2014
*Lasioglossum (Evylaeus) sp.*F5		June 2013<br>June-August 2014<br>June 2016	June 2013<br>May-June 2014<br>June 2015	June 2013<br>June-July 2013
*Lasioglossum (Evylaeus) sp.*F6		June 2014<br>June-August 2016	June 2013<br>June 2016	July 2014
*Lasioglossum (Evylaeus) sp.*F7			July 2014	
*Lasioglossum (Evylaeus) sp.*F8			August 2014	
*Lasioglossum (Evylaeus) sp.*F9			May 2016	
*Lasioglossum (Evylaeus) sp.*F28		August 2014	June-July 2014<br>June 2015	
Lasioglossum (Lasioglossum) anhypops	McGinley, 1986	June-August 2014		June 2013<br>June 2014
Lasioglossum (Lasioglossum) egregium	(Vachal, 1904)	August 2014	June-August 2014<br>June 2015<br>June-July 2016	
Lasioglossum (Lasioglossum) paraforbesii	McGinley, 1986		June 2015	
Lasioglossum (Lasioglossum) sisymbrii	(Cockerell, 1895)	August 2014	June 2015<br>June-July 2016	
Lasioglossum (Leuchalictus) leucozonium	(Schrank, 1781)		June 2015	
Lasioglossum (Sphecodogastra) aberrans	(Crawford, 1903)		June-July 2014	

Species	Authorship	Dates Collected Park Co.	Dates Collected Lewis and Clark Co.	Dates Collected Flathead Co.
Lasioglossum (Sphecodogastra) lusorium	(Cresson, 1872)		July 2015	
Megachilidae				
Anthidiellum (Loyolanthidium) notatum robertsoni	(Cockerell, 1904)		August 2014	
Anthidium (Anthidium) atrifrons	Cresson, 1868	July 2014	August 2014	
Anthidium (Anthidium) clypeodentatum	Swenk, 1914		July-August 2014<br>August 2015<br>July 2016	
Anthidium (Anthidium) formosum	Cresson, 1878		July 2013<br>July 2014<br>June 2015	
Anthidium (Anthidium) mormonum	Cresson, 1878	July 2014	June-July 2013<br>July-August 2014<br>June 2015<br>July 2016	
Anthidium (Anthidium) placitum	Cresson, 1879		August 2015	
Anthidium (Anthidium) tenuiflorae	Cockerell, 1907	August 2016	July-August 2014<br>July 2016	
Anthidium (Anthidium) utahense	Swenk, 1914		July 2013<br>June-August 2014<br>June-August 2015<br>June-August 2016	
Ashmeadiella (Ashmeadiella) gillettei	Titus, 1904		June 2015<br>June 2016	
Ashmeadiella (Ashmeadiella) bucconis	(Say, 1837)		July 2013<br>July-August 2014<br>June-August 2015<br>June-July 2016	
Ashmeadiella (Ashmeadiella) cactorum	(Cockerell, 1897)	July-August 2014<br>July 2015<br>July 2016	July 2013<br>July-August 2014<br>June-August 2015<br>June 2016	

Species	Authorship	Dates Collected Park Co.	Dates Collected Lewis and Clark Co.	Dates Collected Flathead Co.
Ashmeadiella (Ashmeadiella) californica	(Ashmead, 1897)	July 2014<br>June 2015	June-July 2013<br>June-August 2014<br>June-August 2015<br>June-July 2016	
Ashmeadiella (Ashmeadiella) meliloti	(Cockerell, 1897)		August 2014<br>June 2016	
Ashmeadiella (Ashmeadiella) pronitens	(Cockerell, 1906)	July-August 2014<br>June-July 2016		
Chelostoma (Chelostoma) minutum	Crawford, 1916	July 2014		
Coelioxys (Boreocoelioxys) moesta	Cresson, 1864	July-August 2014	August 2014	July 2014
Coelioxys (Boreocoelioxys) porterae	Cockerell, 1900		July-August 2014	July 2014
Coelioxys (Boreocoelioxys) rufitarsis	Smith, 1854		July-August 2014	July 2014
Coelioxys (Coelioxys) sodalis	Cresson, 1878		June 2015	
Coelioxys (Cyrtocoelioxys) modesta	Smith, 1854		August 2015	
Coelioxys (Paracoelioxys) funeraria	Smith, 1854			July 2013<br>June 2015
Coelioxys (Synocoelioxys) alternata	Say, 1837		July 2013<br>July-August 2014<br>June 2015	
Dianthidium (Dianthidium) cressonii	(Dalla Torre, 1896)		August 2016	
Dianthidium (Dianthidium) subparvum	Swenk, 1914		July 2013<br>July-August 2014<br>June-July 2015<br>July-August 2016	
Dianthidium (Dianthidium) ulkei	(Cresson, 1878)		July 2013<br>July-August 2014<br>July-August 2015<br>July-August 2016	
Dioxys productus	(Cresson, 1879)		July 2014	
Heriades (Neotrypetes) carinatus	Cresson, 1864		July 2013<br>July-August 2014<br>July-August 2015<br>June-July 2016	July 2014

Species	Authorship	Dates Collected Park Co.	Dates Collected Lewis and Clark Co.	Dates Collected Flathead Co.
Heriades (Neotrypetes) cressoni	Michener, 1938	August 2014	July 2013<br>July-August 2014<br>July-August 2015<br>June-August 2016	
Heriades (Neotrypetes) variolosa	(Cresson, 1872)		July 2013<br>July-August 2014<br>July-August 2015	July 2013
Hoplitis (Alcidamea) albifrons argentifrons	(Cresson, 1864)	July 2013<br>June-August 2014<br>June-July 2015<br>June-August 2016	June-July 2013<br>June-August 2014<br>June 2015<br>June-July 2016	July 2013<br>July 2014<br>June-July 2015
Hoplitis (Alcidamea) fulgida fulgida	(Cresson, 1864)	June-July 2013<br>July-August 2014<br>June-August 2015<br>June-August 2016	June 2013<br>June 2014	July 2013<br>July 2014
Hoplitis (Alcidamea) grinnelli	(Cockerell, 1910)		June-July 2014<br>June 2015	
Hoplitis (Alcidamea) hypocrita	(Cockerell, 1906)		June-July 2013<br>May-July 2014<br>June-July 2015<br>June-July 2016	
Hoplitis (Alcidamea) producta	(Cresson, 1864)	August 2014<br>June-July 2015	July-August 2014<br>June 2015<br>June 2016	July 2013
Hoplitis (Alcidamea) truncata	(Cresson, 1878)		June-July 2013<br>June-August 2014<br>June-August 2015<br>June-July 2016	
Hoplitis (Formicapis) robusta	(Nylander, 1848)	July 2014<br>June-July 2016		
Megachile (Argyropile) parallela	Smith, 1853		July-August 2014<br>July 2015<br>July-August 2016	

Species	Authorship	Dates Collected Park Co.	Dates Collected Lewis and Clark Co.	Dates Collected Flathead Co.
Megachile (Chelostomoides) campanulae	(Robertson, 1903)		June-July 2015<br>July 2016	
Megachile (Chelostomoides) angelarum	Cockerell, 1902	August 2016	July-August 2014<br>July 2016	
Megachile (Eutricharaea) apicalis	Spinola, 1808	July 2016	July 2013<br>July-August 2014<br>June-August 2015<br>June-August 2016	
Megachile (Eutricharaea) rotundata	(Fabricius, 1793)		July-August 2015	
Megachile (Litomegachile) brevis	Say, 1837		July-August 2014<br>June 2015	
Megachile (Litomegachile) onobrychidis	Cockerell, 1905		June-August 2014	
Megachile (Litomegachile) texana	Cresson, 1878		July-August 2014	
Megachile (Megachile) lapponica	Thomson, 1872	July 2013<br>July-August 2014<br>June-July 2015<br>June-August 2016	June 2015	July 2013<br>July 2014<br>June 2015
Megachile (Megachile) montivaga	Cresson, 1878	June 2016	July 2014<br>July-August 2015<br>July 2016	
Megachile (Megachile) relativa	Cresson, 1878	July 2013<br>July-August 2014<br>July-August 2015<br>July-August 2016	June-August 2014<br>June 2015<br>July-August 2016	July 2013<br>July 2014<br>June-July 2015
Megachile (Megachiloides) subnigra	Cresson, 1879		June 2015	
Megachile (Megachiloides) wheeleri	Mitchell, 1927		August 2014	
Megachile (Sayapis) fidelis	Cresson, 1878		July-August 2014<br>July-August 2015<br>July-August 2016	
Megachile (Sayapis) pugnata	Say, 1837	July 2013<br>July-August 2014<br>July 2015<br>June-August 2016	July 2013<br>June-August 2014<br>June-August 2015<br>June-August 2016	June 2014

Species	Authorship	Dates Collected Park Co.	Dates Collected Lewis and Clark Co.	Dates Collected Flathead Co.
Megachile (Xanthosarus) frigida	Smith, 1853	July 2013<br>July-August 2014<br>June-August 2015<br>June-August 2016	June-July 2013<br>June-August 2014<br>June-August 2015<br>June-August 2016	July 2013<br>July-August 2014<br>July 2015
Megachile (Xanthosarus) gemula	Cresson, 1878	July-August 2014<br>June 2015<br>July 2016	June 2013<br>June-July 2014	July 2013<br>July 2014
Megachile (Xanthosarus) latimanus	Say, 1823		July 2015<br>August 2016	
Megachile (Xanthosarus) melanophaea	Smith, 1853	July-August 2014<br>June-July 2015<br>June-July 2016	June 2013<br>June-July 2014<br>June 2015	
Megachile (Xanthosarus) perihirta	Cockerell, 1898	July-August 2014<br>July-August 2016	June-July 2013<br>June-August 2014<br>June-August 2015<br>June-August 2016	July 2014
Osmia (Cephalosmia) californica	Cresson, 1864		June-July 2013<br>May-June 2014<br>June 2015<br>May-June 2016	
Osmia (Cephalosmia) marginipennis	Cresson, 1878		May-June 2014<br>June 2015<br>May 2016	
Osmia (Cephalosmia) montana montana	Cresson, 1864	July 2014<br>June-July 2015<br>June 2016	June 2013<br>May-July 2014<br>June 2015<br>May-June 2016	
Osmia (Cephalosmia) subaustralis	Cockerell, 1900	August 2014<br>June-August 2015<br>June 2016	June 2016	
Osmia (Hapsidosmia) iridis	Cockerell and Titus, 1902		June 2013<br>May-June 2014<br>June 2015	

Species	Authorship	Dates Collected Park Co.	Dates Collected Lewis and Clark Co.	Dates Collected Flathead Co.
Osmia (Helicosmia) coloradensis	Cresson, 1878	July 2014<br>June-August 2016	June-July 2013<br>May-July 2014<br>June 2015<br>May-July 2016	June 2013<br>July 2014<br>June 2015
Osmia (Helicosmia) texana	Cresson, 1872		July 2013<br>July-August 2014<br>July 2015	
Osmia (Melanosmia) aff.*albolateralis*	Cockerell, 1906		July 2014	
Osmia (Melanosmia) aff.*grindeliae*	Cockerell, 1900		July 2014	
Osmia (Melanosmia) aff.*paradisica*	Sandhouse, 1924	July-August 2014<br>August 2015<br>August 2016	July 2014	July 2014
Osmia (Melanosmia) aff.*pusilla*	Cresson, 1864	July 2014		
Osmia (Melanosmia) albolateralis	Cockerell, 1906	June-July 2013<br>June-August 2014<br>June-July 2015<br>June-July 2016	June-July 2013<br>May-August 2014<br>June 2015<br>June-July 2016	July 2013<br>June-July 2014<br>June 2015
Osmia (Melanosmia) atrocyanea	Cockerell, 1897	June 2016	June 2013<br>June-July 2014<br>June 2015<br>June-July 2016	
Osmia (Melanosmia) brevis	Cresson, 1864	June 2014<br>June 2015<br>June 2016	June-July 2013<br>May-June 2014<br>June 2015	
Osmia (Melanosmia) bruneri	Cockerell, 1897		June 2013<br>June 2014<br>June 2015<br>June-July 2016	
Osmia (Melanosmia) bucephala	Cresson, 1864	July 2013<br>July 2014<br>June-July 2015<br>June 2016	June 2013<br>June 2015	July 2013<br>July 2014
Osmia (Melanosmia) cyanella	Cockerell, 1897			July 2014

Species	Authorship	Dates Collected Park Co.	Dates Collected Lewis and Clark Co.	Dates Collected Flathead Co.
Osmia (Melanosmia) densa	Cresson, 1864	July 2013<br>July 2015<br>June 2016	June-July 2013<br>May-July 2014<br>June 2015<br>May-June 2016	June-July 2015
Osmia (Melanosmia) ednae	Cockerell, 1907	July 2013<br>June-July 2014		
Osmia (Melanosmia) grindeliae	Cockerell, 1910	July 2014	June-July 2014	June 2014
Osmia (Melanosmia) inermis	(Zetterstedt, 1838)	July 2014<br>June 2015<br>June 2016	June-July 2014	July 2014
Osmia (Melanosmia) integra	Cresson, 1878		May 2016	
Osmia (Melanosmia) juxta	Cresson, 1864	June-August 2014<br>June 2015<br>June-August 2016	June-July 2013<br>May-July 2014<br>June-July 2015<br>May-July 2016	July 2013<br>July 2014<br>June 2015
Osmia (Melanosmia) kincaidii	Cockerell, 1897		June 2013<br>June 2014<br>June 2015	
Osmia (Melanosmia) longula	Cresson, 1864	July 2013	May-July 2014	
Osmia (Melanosmia) malina	Cockerell, 1909		July 2014	
Osmia (Melanosmia) nigrifrons	Cresson, 1878	July-August 2014<br>June 2016	June 2013<br>June-July 2014<br>June 2015<br>May 2016	
Osmia (Melanosmia) nigriventris	(Zetterstedt, 1838)	July 2014<br>July 2016		
*Osmia (Melanosmia) odontogaster gr.sp.*1		June 2013	June 2014	
*Osmia (Melanosmia) odontogaster gr.sp.*2		August 2014	June 2016	
Osmia (Melanosmia) paradisica	Sandhouse, 1924	August 2014<br>July-August 2015<br>June 2016		

Species	Authorship	Dates Collected Park Co.	Dates Collected Lewis and Clark Co.	Dates Collected Flathead Co.
Osmia (Melanosmia) pentstemonis	Cockerell, 1906	June 2013<br>June-August 2014<br>June 2016	July 2014	
Osmia (Melanosmia) phaceliae	Cockerell, 1907	July 2014<br>June-July 2016	July 2014<br>June 2015<br>June 2016	
Osmia (Melanosmia) physariae	Cockerell, 1907	June 2014		
Osmia (Melanosmia) pikei	Cockerell, 1907	June 2014		
Osmia (Melanosmia) proxima	Cresson, 1864	August 2014		
Osmia (Melanosmia) pusilla	Cresson, 1864	July-August 2014<br>August 2016	June 2013<br>June-August 2014<br>June 2015<br>June-July 2016	July 2014
Osmia (Melanosmia) sculleni	Sandhouse, 1939	June 2014		
Osmia (Melanosmia) simillima	Smith, 1853		June 2013<br>May-June 2014<br>June	
Osmia (Melanosmia) sp.3				July 2014
Osmia (Melanosmia) sp.9			June 2013<br>June 2016	
Osmia (Melanosmia) tersula	Cockerell, 1912	June 2013<br>June-July 2014<br>June 2015<br>June 2016	May 2016	
Osmia (Melanosmia) trevoris	Cockerell, 1897		June-July 2013<br>June-August 2014<br>June 2015<br>June-July 2016	
Osmia (Melanosmia) tristella	Cockerell, 1897	July 2014<br>July 2015<br>June 2016	June-July 2014<br>June 2015<br>June-July 2016	June-July 2014

Species	Authorship	Dates Collected Park Co.	Dates Collected Lewis and Clark Co.	Dates Collected Flathead Co.
Osmia (Osmia) lignaria propinqua	Cresson, 1864	July 2014<br>June 2016	June 2015	
Stelis (Stelis) aff. *permaculata*	Cockerell, 1898		July-August 2014	
Stelis (Stelis) calliphorina	(Cockerell, 1911)		July-August 2014<br>June-July 2015	
Stelis (Stelis) callura	Cockerell, 1925		June 2014	
Stelis (Stelis) carnifex	Cockerell, 1911	June 2015	June 2014	
*Stelis (Stelis) foederalis gr.sp.*2		July 2016		
*Stelis (Stelis) foederalis gr.sp.*6		July 2014	June 2015	July 2013
*Stelis (Stelis) foederalis gr.sp.*7		July 2016		
*Stelis (Stelis) foederalis gr.sp.*8		August 2014		
Stelis (Stelis) montana	Cresson, 1864	June-July 2013<br>July-August 2014	June-July 2013<br>July-August 2014<br>June 2015<br>June-July 2016	July 2013
Stelis (Stelis) monticola	Cresson, 1878	June 2015	June 2013<br>June 2014<br>June 2015	
Stelis (Stelis) nitida	Cresson, 1878	July 2013		
Stelis (Stelis) permaculata	Cockerell, 1898		July 2013<br>August 2014<br>July 2015	
Apidae				
Anthophora (Clisodon) terminalis	Cresson, 1869	June 2015<br>July 2016	July 2013<br>June-July 2014<br>June-July 2015<br>June-July 2016	
Anthophora (Lophanthophora) pacifica	Cresson, 1878		June 2015	
Anthophora (Lophanthophora) ursina	Cresson, 1869		June 2013<br>May-June 2014<br>June 2015<br>May-June 2016	

Species	Authorship	Dates Collected Park Co.	Dates Collected Lewis and Clark Co.	Dates Collected Flathead Co.
Anthophora (Melea) bomboides	Kirby, 1838		July 2014<br>June-July 2015<br>May-June 2016	
Anthophora (Mystacanthophora) urbana	Cresson, 1878		July 2013<br>July-August 2014<br>July 2016	
Apis mellifera	Linnaeus, 1758	August 2014	June 2013<br>May-August 2014<br>June-July 2015<br>June 2016	
Bombus (Bombus) occidentalis	Greene, 1858	July-August 2014<br>July-August 2016		July 2013<br>July-August 2014
Bombus (Cullumanobombus) griseocollis	(De Geer, 1773)		July 2013<br>August 2014<br>July-August 2015<br>July-August 2016	
Bombus (Cullumanobombus) rufocinctus	Cresson, 1863	July 2013<br>July-August 2014<br>July-August 2015<br>July-August 2016	July 2013<br>July-August 2014<br>June-August 2016	July 2013<br>July 2014
Bombus (Psithyrus) flavidus	Eversmann, 1852	July-August 2014<br>August 2016		June-July 2013<br>July 2014
Bombus (Psithyrus) insularis	(Smith, 1861)	July-August 2014<br>July-August 2015<br>July-August 2016	August 2014<br>June-August 2015<br>July 2016	July 2013<br>July 2014
Bombus (Psithyrus) suckleyi	Greene, 1860			July 2013
Bombus (Pyrobombus) bifarius	Cresson, 1878	June-July 2013<br>June-August 2014<br>June-August 2015<br>June-August 2016	July 2013<br>June-August 2014<br>June-August 2015<br>May-August 2016	June-July 2013<br>June-August 2014<br>June-July 2015

Species	Authorship	Dates Collected Park Co.	Dates Collected Lewis and Clark Co.	Dates Collected Flathead Co.
Bombus (Pyrobombus) centralis	Cresson, 1864	June 2013<br>July-August 2014<br>July-August 2016	July 2013<br>June-August 2014<br>June-August 2015<br>June-August 2016	July 2013<br>June-July 2014
Bombus (Pyrobombus) flavifrons	Cresson, 1863	June-July 2013<br>June-August 2014<br>June-August 2015<br>June-August 2016	July 2013<br>June-August 2014<br>July 2015<br>June-August 2016	July 2013<br>June-July 2014<br>June 2015
Bombus (Pyrobombus) huntii	Greene, 1860	August 2014<br>August 2016	July 2013<br>July-August 2014<br>June-August 2015<br>June-August 2016	
Bombus (Pyrobombus) melanopygus	Nylander, 1848	July 2013<br>July-August 2014<br>July-August 2016	July 2016	June-July 2013<br>June-July 2014<br>July 2015
Bombus (Pyrobombus) mixtus	Cresson, 1878	July 2013<br>June-August 2014<br>July 2015<br>July-August 2016	July 2016	June-July 2013<br>June-July 2014<br>July 2015
Bombus (Pyrobombus) sitkensis	Nylander, 1848			July 2013<br>June-July 2014<br>June-July 2015
Bombus (Pyrobombus) vagans	Smith, 1854	August 2016		July 2013<br>July 2014
Bombus (Subterraneobombus) appositus	Cresson, 1878	July-August 2014<br>August 2015<br>June-August 2016	July 2013<br>June-August 2014<br>July-August 2015<br>June-August 2016	July 2013<br>July 2014
Bombus (Subterraneobombus) borealis	Kirby, 1837		August 2014<br>August 2016	
Bombus (Thoracobombus) californicus	Smith, 1854			July 2014

Species	Authorship	Dates Collected Park Co.	Dates Collected Lewis and Clark Co.	Dates Collected Flathead Co.
Bombus (Thoracobombus) fervidus	(Fabricius, 1798)	August 2014<br>August 2016	July 2013<br>August 2014<br>June-August 2015<br>June-August 2016	
Ceratina (Zadontomerus) nanula	Cockerell, 1897	June-July 2014<br>June 2015	June-July 2013<br>June-August 2014<br>June-July 2015<br>June-July 2016	July 2014
Ceratina (Zadontomerus) neomexicana	Cockerell, 1901	June 2016	June-July 2013<br>May-August 2014<br>June-July 2015<br>May-August 2016	
Diadasia (Coquillettapis) diminuta	(Cresson, 1878)		June 2014	
Epeolus sp.			August 2014	
Eucera (Synhalonia) edwardsii	(Cresson, 1878)	June 2016	June-July 2013<br>June-July 2014<br>June 2015<br>June 2016	
Eucera (Synhalonia) frater	(Cresson, 1878)	June 2016	June 2013<br>May-June 2014<br>June 2016	
Eucera (Synhalonia) fulvitarsis	(Cresson, 1878)		June 2013<br>June 2014<br>June 2016	
Melecta (Melecta) pacifica fulvida	Cresson, 1878		June 2014<br>June 2015	
Melecta (Melecta) separata	Cresson, 1879		May 2014	
Melissodes (Eumelissodes) confusa	Cresson, 1878		August 2014<br>August 2015	
Melissodes (Eumelissodes) coreopsis	Robertson, 1905		July 2014<br>August 2015<br>August 2016	
Melissodes (Eumelissodes) hymenoxidis	Cockerell, 1906		July 2014	

Species	Authorship	Dates Collected Park Co.	Dates Collected Lewis and Clark Co.	Dates Collected Flathead Co.
Melissodes (Eumelissodes) microstictus	Cockerell, 1905	August 2014<br>August 2016	July 2013<br>July-August 2014<br>July 2015<br>June-August 2016	
Melissodes (Eumelissodes) utahensis	LaBerge, 1961		August 2014	
Melissodes (Heliomelissodes) rivalis	Cresson, 1872		July 2014<br>July 2015<br>July 2016	
*Melissodes sp.*F1			August 2015	
*Melissodes sp.*F2			August 2016	
Melissodes unk1			August 2014	
Nomada edwardsii	Cresson, 1878		June 2013<br>May 2014<br>June 2015<br>June 2016	
*Nomada sp.*F1			June 2014<br>June 2015<br>May 2016	
*Nomada sp.*F2			June 2014<br>June 2015<br>June 2016	
*Nomada sp.*F3		July 2014	June 2013<br>June-July 2014<br>June 2015<br>May 2016	
*Nomada sp.*F4			June 2014	
*Nomada sp.*F5			June 2013	
*Nomada sp.*F6		July 2014		
*Nomada sp.*F7			June 2013<br>May-June 2014	
*Nomada sp.*F8				July 2013
Triepeolus paenepectoralis	Viereck, 1905		August 2014	
